# A Tale of a Life-Threatening Twist: A Rare Case of Transverse Colonic Volvulus in an Infant

**DOI:** 10.7759/cureus.96233

**Published:** 2025-11-06

**Authors:** Usman A Dar, Paul P Elbayeh, Jessica M Ngo, Morgan Jones, Chetan Moorthy

**Affiliations:** 1 Department of Radiology, Texas Tech University Health Sciences Center Paul L. Foster School of Medicine, El Paso, USA; 2 Department of Pathology, Texas Tech University Health Sciences Center Paul L. Foster School of Medicine, El Paso, USA; 3 Division of Imaging, El Paso Children’s Hospital, El Paso, USA

**Keywords:** acute abdomen in children, barium enema, pediatric volvulus, radiologic diagnosis of volvulus, transverse colon volvulus

## Abstract

Transverse colon volvulus (TCV) secondary to intestinal malrotation is exceptionally rare in pediatric patients, frequently causing delay in diagnosis and severe complications. Prompt recognition guided by diagnostic imaging is critical for improving outcomes in affected children.

A previously healthy nine-month-old male presented acutely with significant abdominal distension, metabolic acidosis, and septic shock. Initial plain radiography indicated colonic obstruction, and subsequent ultrasound was equivocal in elucidating the cause of obstruction. Eventually, a contrast-enhanced barium enema confirmed the cause of obstruction as colonic volvulus, demonstrating a characteristic "beaked" appearance within the transverse colon. Subsequent emergent laparotomy revealed extensive bowel necrosis due to volvulus-induced ischemia caused by intestinal malrotation. Timely surgical management included ileocolectomy, temporary open abdomen management, and successful staged reconstruction, resulting in complete patient recovery.

This case highlights the importance of considering uncommon, acutely life-threatening etiologies of disease processes in the pediatric population such as colonic volvulus. It also signifies the indispensable role of dynamic contrast-enhanced imaging studies that not only facilitate the diagnosis of colonic volvulus in the pediatric population but also allow for timely surgical management, and thus significantly reduce morbidity and mortality.

## Introduction

Pediatric colonic volvulus is a rare cause of intestinal obstruction, frequently posing diagnostic challenges to clinicians and radiologists due to nonspecific clinical presentations and seldom subtle initial imaging findings [1]. Transverse colon volvulus (TCV) is even more exceedingly uncommon, with up to 63 reported cases between 1932 and 2021, according to the world literature review conducted by Huerta et al. [1]. Therefore, we estimate fewer than 100 reported cases of pediatric TCV at the time of this study.

Colonic volvulus typically arises in segments of the colon endowed with a more extensive mesentery, which confers increased mobility. The mechanism of volvulus involving the transverse colon is similar to that of other large-bowel segments [2]. In native anatomy, the ascending and descending colon are fused to the retroperitoneum, whereas the transverse and sigmoid colon maintain considerable freedom of movement. In TCV, abnormal mesenteric elongation predisposes the colon to axial rotation, resulting in luminal obstruction and ischemia [2]. Congenital anomalies (e.g., malrotation, Hirschsprung’s disease) serve as additional risk factors for TCV, as they promote abnormal fixation and increased mobility, thereby significantly elevating the risk of volvulus and subsequent vascular compromise [2].

TCV is a surgical emergency due to its high potential for bowel ischemia and necrosis, necessitating timely diagnosis and intervention [2]. Multimodal imaging plays a crucial role in prompt diagnosis. In the pediatric population, abdominal plain films are often highly useful for initial screening of bowel obstruction [3]. Ultrasonography, given its accessibility, portability, and lack of ionizing radiation, is frequently the first dynamic imaging modality used both to investigate the etiology of bowel obstruction and to rule out other abdominal pathologies [3]. A barium enema dynamic study is considered highly effective in confirming the diagnosis of colonic volvulus and may occasionally be therapeutic [4]. Although computed tomography (CT) is a mainstay in diagnosing volvulus in adults, it is not preferred in children due to significant radiation exposure [5]. Magnetic resonance (MR) imaging is not typically used for diagnosing pediatric colonic volvulus, but can be valuable in cases with equivocal barium enema results or when fluoroscopy is unavailable [6].

This report presents an illustrative case of TCV secondary to intestinal malrotation. It underscores the importance of heightened clinical awareness of malrotation as a significant underlying risk factor for pediatric colonic volvulus. Early identification through appropriate imaging modalities is critical, as timely diagnosis significantly impacts management and outcomes, reducing the morbidity associated with diagnostic delays and ischemic complications.

## Case presentation

A previously healthy, nine-month-old male presented to the children’s hospital emergency department with one day of progressive feeding intolerance, lethargy, and multiple episodes of non-bilious emesis occurring one to two hours after formula feeds. Caregivers reported firmer stools in recent days without melena or hematochezia. On arrival, vital signs were notable only for a heart rate at the upper limit of normal (156 beats per minute; age reference 100-160 bpm). Initial laboratory studies demonstrated leukocytosis with a mildly increased neutrophilic fraction and anemia. On examination, the infant was arousable with marked abdominal distension and no voluntary or involuntary guarding.

**Figure 1:**
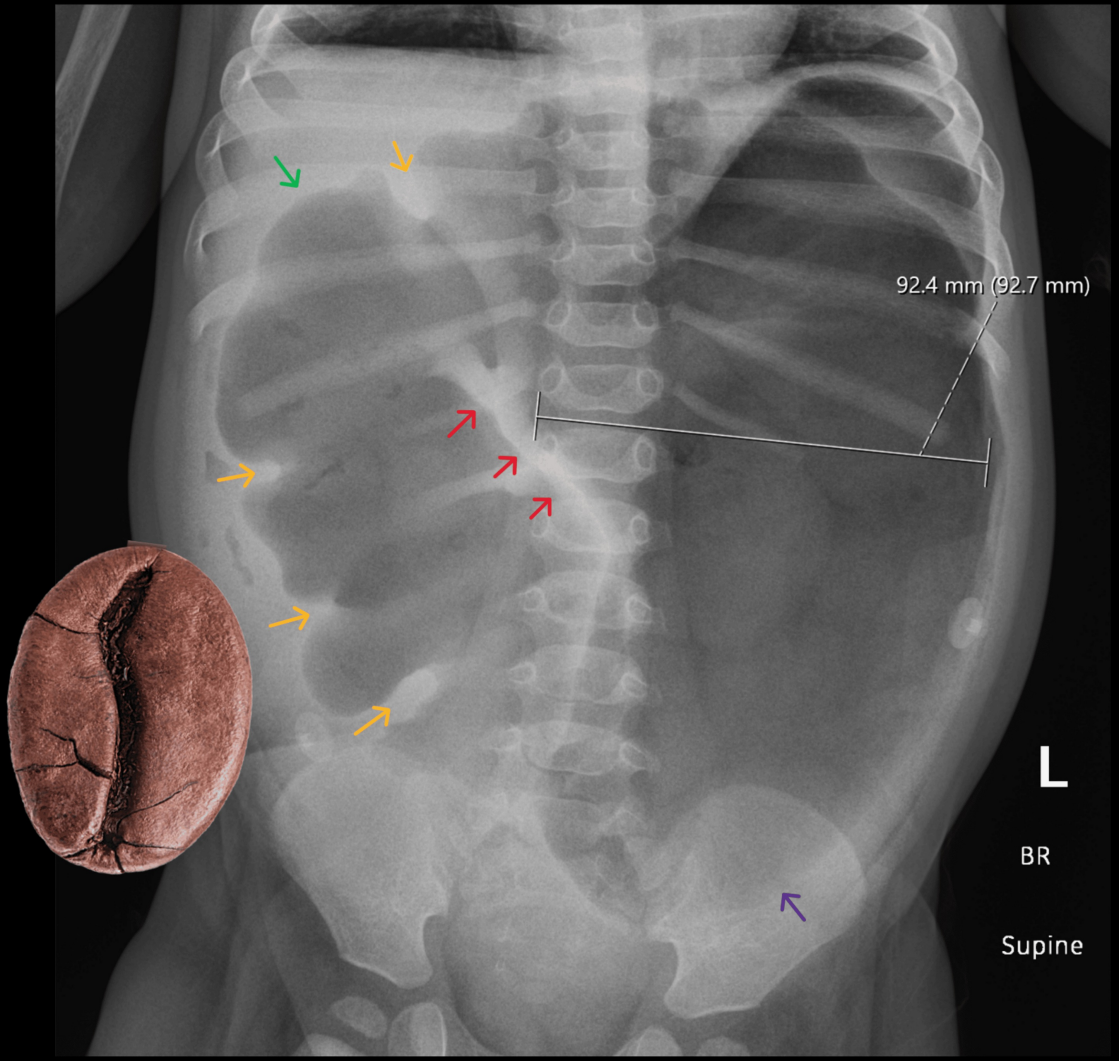
Abdominal radiograph (AP view) demonstrates marked gaseous distension of the large bowel, measuring up to 9.2 cm (on-image calipers). The dilated bowel is identified as the proximal segment of the transverse colon, as indicated by characteristic colonic haustral folds (yellow arrows) and the typical course of the bowel from the hepatic flexure (green arrow). An obliquely oriented “white stripe” sign is seen (red arrows), representing apposed walls of a gas-filled colonic loop resulting from a bowel twist. Overall, the distended proximal transverse colon segment demonstrates a coffee-bean configuration, with its apex directed toward the left iliac fossa (purple arrow), highly concerning for volvulus. Note that this orientation should not be confused with the classic “coffee-bean (omega)” sign seen typically in cases of sigmoid volvulus, in which the apex usually points to the right upper quadrant. AP = anteroposterior

**Figure 2:**
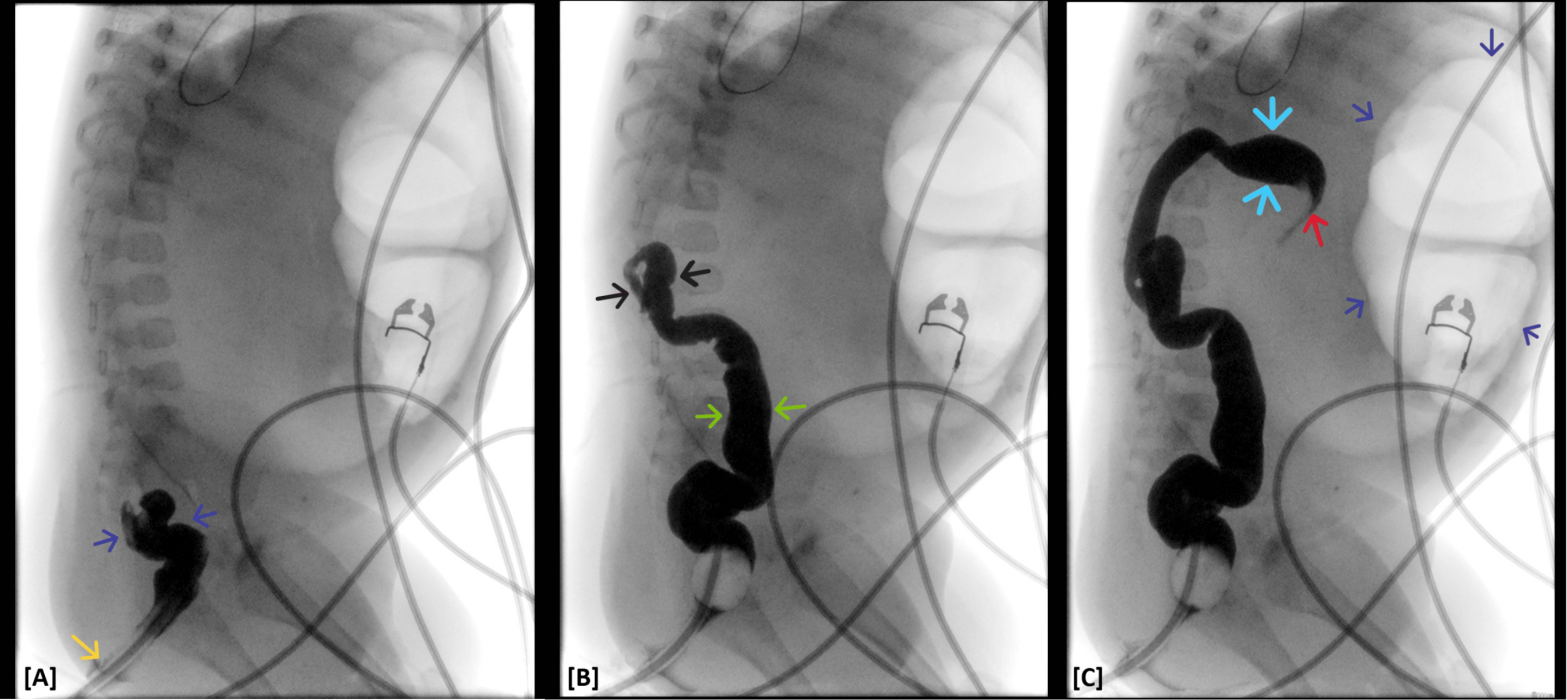
Dynamic barium enema abdominal fluoroscopy, right lateral view [A]: Thin barium contrast enema administration via a 16 French rectal catheter (yellow arrow) outlining a decompressed rectosigmoid segment (blue arrows) of the bowel. [B]: Dynamic subsequent contrast opacification of a small caliber descending (green arrows) and distal transverse (black arrows) colon. [C]: “Beaked” tapering of the barium enema contrast opacification with an abrupt cut-off at the level of the mid-transverse colon (blue arrows), corresponding to the point of high-grade mechanical bowel obstruction (red arrow). Note the extensive gaseous distension of the large bowel (purple arrows) proximal to the point of obstruction.

**Figure 3:**
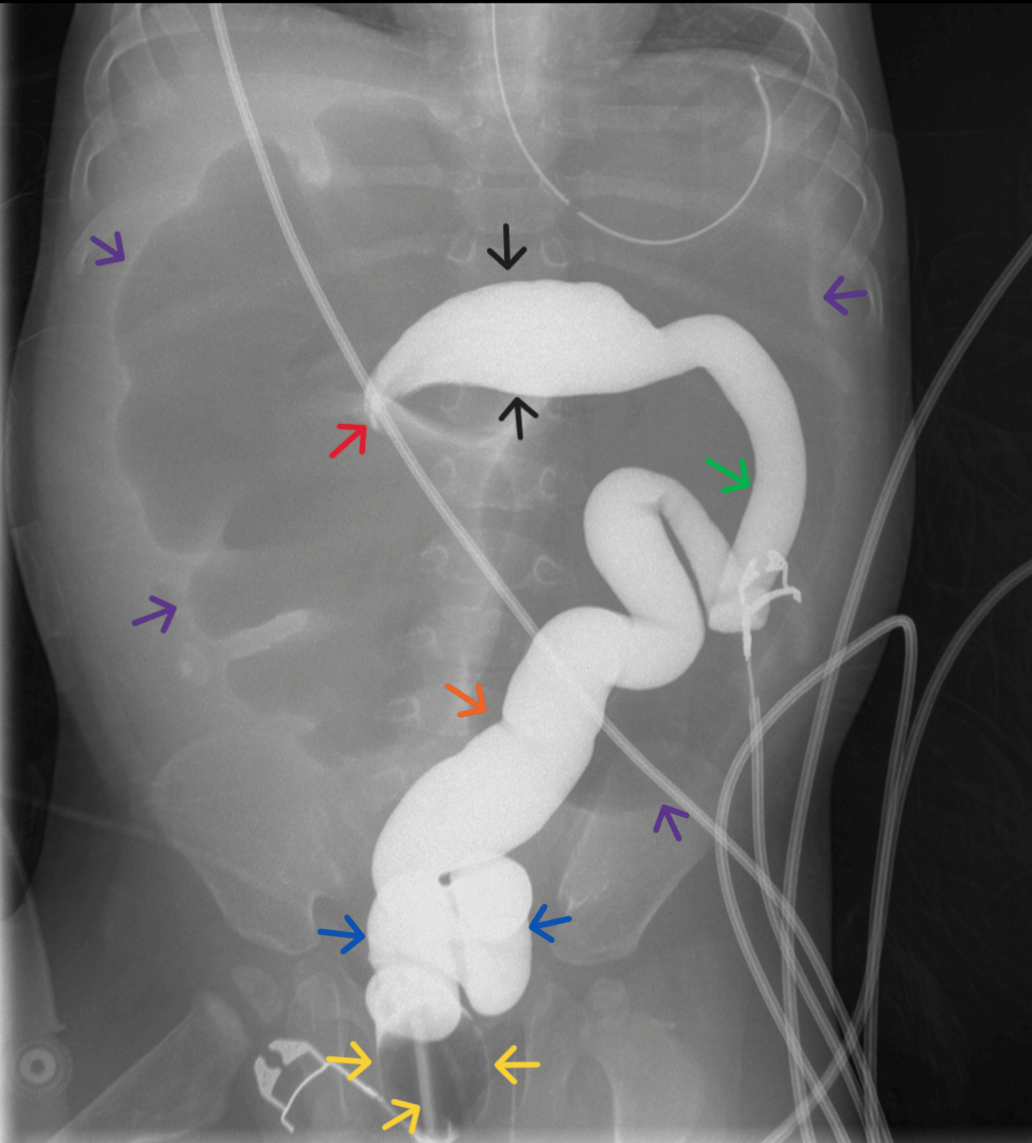
High-resolution spot film (AP view) from a dynamic fluoroscopy-assisted barium enema study shows evidence of a tapered obstruction at the level of the mid-transverse colon (black arrows), with a beaked appearance (red arrow), corresponding to a point of high-grade mechanical bowel obstruction. The transition zone (red arrow) projects over the gas-filled proximal colonic loops (purple arrows). The rectal catheter and inflated balloon (yellow arrows) are positioned within the rectum (blue arrows), and there is adequate contrast opacification of the sigmoid (orange arrow) and descending (green arrow) colon. AP = anteroposterior

Given concern for bowel obstruction, urgent plain abdominal radiography was obtained. Imaging demonstrated significant gaseous distension of large-bowel loops, raising suspicion for mechanical large-bowel obstruction (Figure 1), initially thought to be at the sigmoid colon. The distribution and configuration of the dilated loops raised concern that the transition point was localized to the transverse colon rather than the sigmoid, which pediatric radiology communicated to the surgical team. In consultation with pediatric radiology and pediatric surgery, a contrast barium enema was promptly performed to delineate the etiology and localize the transition point for operative planning. Contrary to the preliminary impression, the enema showed a normal sigmoid colon and a tapered “beaked” narrowing at the transverse colon, confirming transverse colonic volvulus (Figures 2, 3). An attempt at nonoperative detorsion during the enema was unsuccessful.

While preparations for operative intervention were underway, repeat clinical and laboratory evaluation indicated rapid deterioration with profound hematologic and metabolic derangements (Table 1), including worsening leukocytosis, primary metabolic acidosis, hyperkalemia, biochemical evidence of tissue hypoperfusion, and moderate hypoxemia. Taken together, these findings were consistent with severe metabolic decompensation characterized by lactic acidosis, hypoxemic respiratory failure, electrolyte abnormalities, and a systemic inflammatory response, most likely secondary to acute intestinal ischemia.

**Table 1:** Representative laboratory findings on the day of presentation demonstrating severe metabolic acidosis with lactic acidosis, hypoxemia, electrolyte derangements, and hematologic abnormalities consistent with systemic ischemia ABG = arterial blood gas; pH = hydrogen ion concentration; HCO₃⁻ = serum bicarbonate; PaO₂ = partial pressure of arterial oxygen; WBC = white blood cell count; INR = international normalized ratio; aPTT = activated partial thromboplastin time.

Parameter	Value	Reference Range
Arterial blood gas		
pH	7.19	7.35 - 7.45
HCO_3_^-^ (mmol/L)	15.5	22 - 26
Base excess (mmol/L)	-12.2	-2 - +2
Lactate (mmol/L)	5.86	< 2.0
PaO_2_ (mmHg)	61.0	80 - 100
Oxygen saturation (%)	85.1	> 95
Electrolytes and glucose		
Potassium (mmol/L)	6.67	3.5 - 5.7 (infant)
Sodium (mmol/L)	134	135 - 145
Glucose (mg/dL)	236	70 - 140
Hematology		
Hematocrit (%)	44.5	30 - 40
WBC (x10^3^/µL)	24.3	6 - 18 (9 mo)
Neutrophils	High	1.5 - 8.5
Bands	High	< 0.5
Lymphocytes	Low	50 - 70% (9 mo)
Coagulation		
Protime (sec)	16.4	11 - 15
INR	1.3	0.9 - 1.1
aPTT (sec)	37.2	28 - 40

**Figure 4:**
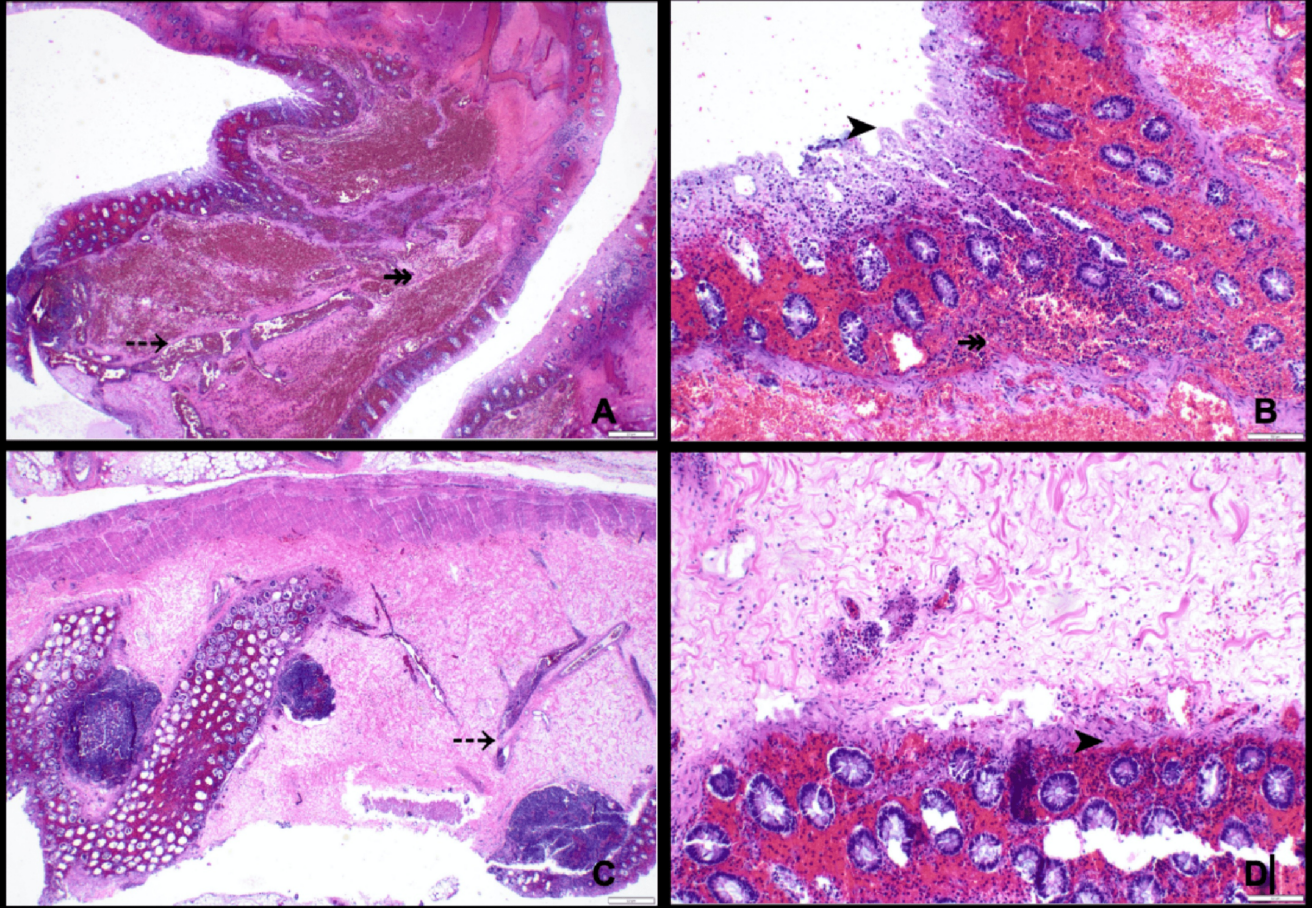
H&E-stained sections (A-D) of the proximal large bowel segment demonstrate colonic mucosa with ischemic-type necrosis (➤), vascular congestion (⤏), and transmural hemorrhage (↠) H&E = hematoxylin and eosin

Subsequent emergency exploratory laparotomy confirmed transverse colonic volvulus secondary to intestinal malrotation, with extensive ischemic injury involving the distal ileum and the ascending and transverse colon. The patient underwent ileocolectomy and resection of the ischemic bowel segments in conjunction with a Ladd procedure. Because of ongoing critical illness, the need for continued resuscitation, and concern for bowel and mesenteric edema, the abdomen was managed with a temporary negative-pressure dressing (ABThera), and the intestinal tract was intentionally left in discontinuity. Gross pathologic examination of the resected segment demonstrated features of ischemic injury, including dusky discoloration of the colonic wall and focal mucosal sloughing. Histopathologic evaluation (Figures 4A-4D) confirmed ischemic-type necrosis with marked vascular congestion and transmural hemorrhage, correlating with the grossly compromised transverse colon and supporting the diagnosis of ischemic colitis secondary to volvulus.

**Figure 5:**
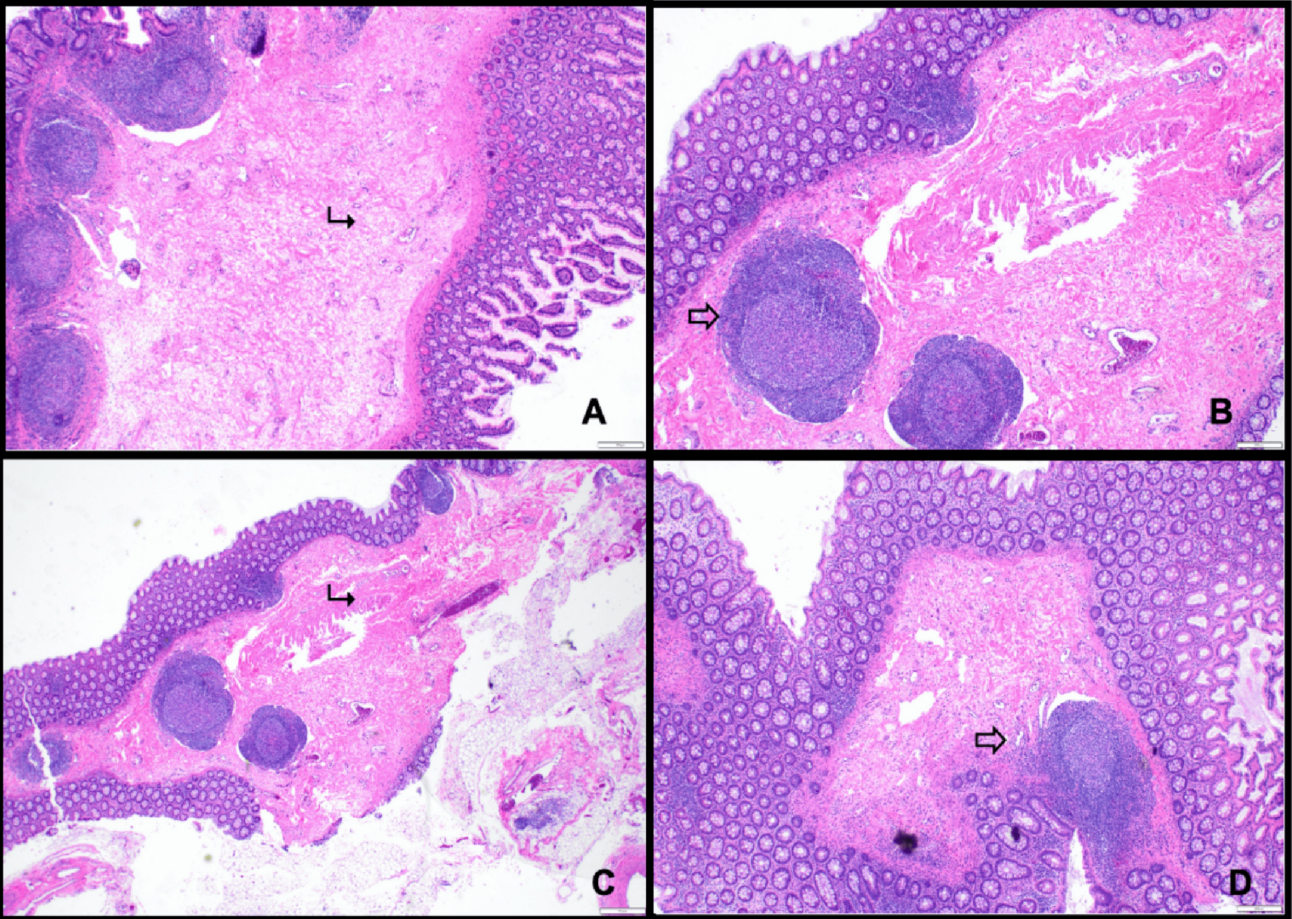
H&E-stained low-power sections of the distal ileum (A) and proximal large bowel segments (B-D) demonstrate viable small bowel and colonic mucosa with chronic inflammation (⇨) and edematous changes (↳). H&E = hematoxylin and eosin

After hemodynamic stabilization in the intensive care unit, the patient returned to the operating room on postoperative day 3 for planned re-exploration, removal of the negative-pressure dressing, restoration of intestinal continuity, and abdominal closure. The small bowel was viable without new ischemia or volvulus. A hand-sewn end-to-end ileocolic anastomosis was created after limited additional resection to relieve tension and address caliber discrepancy, followed by uncomplicated fascial closure. Histopathologic examination of the newly resected margins demonstrated viable small-bowel and colonic tissue with chronic inflammation and edema, consistent with interval reperfusion and healing (Figures 5A-5D), thereby further corroborating successful intervention.

**Figure 6:**
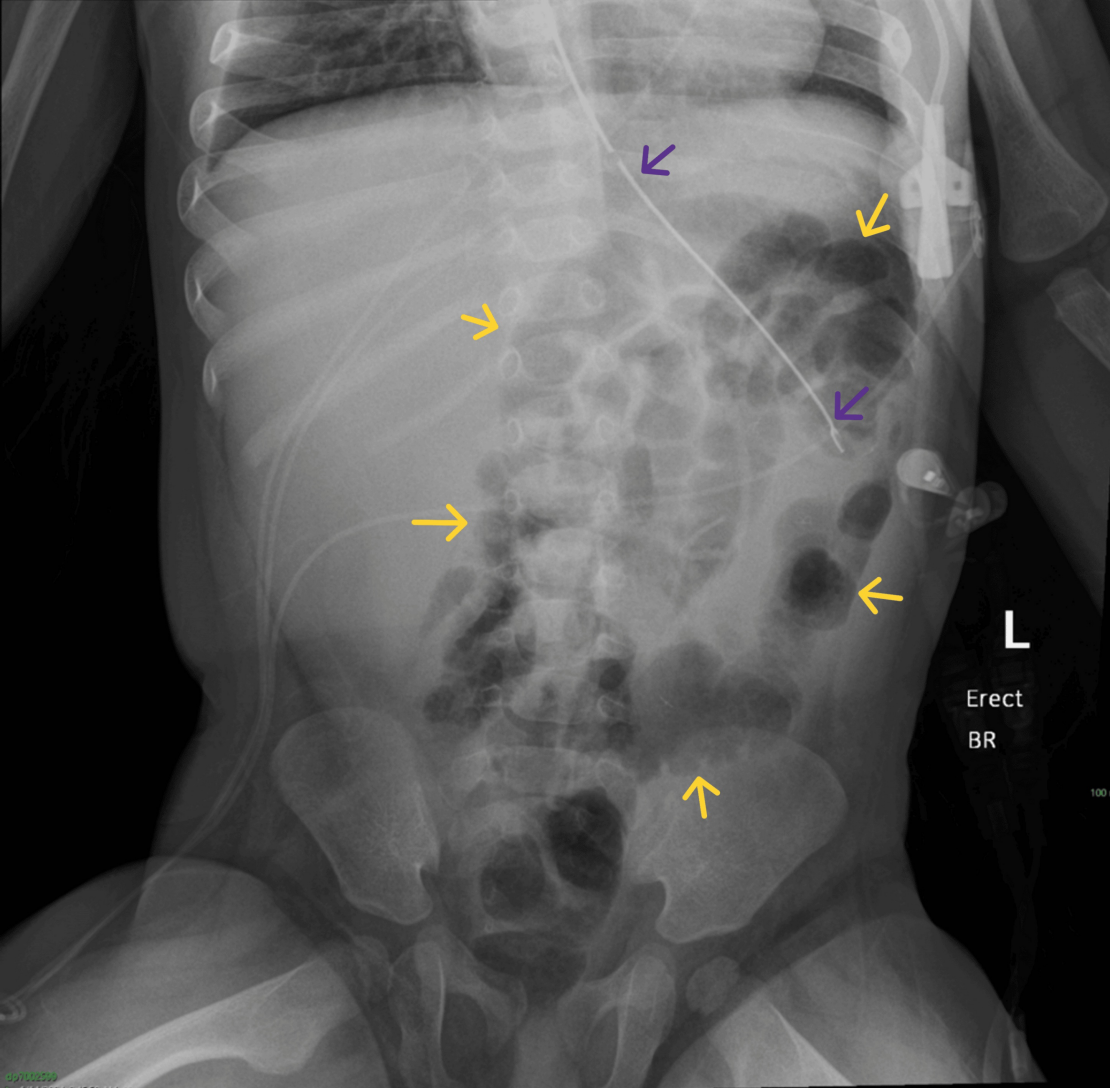
One-week postintervention abdominal radiograph, AP view: complete resolution of previously seen abnormal large bowel gaseous distension. There is a normal, non-obstructive large bowel gas pattern (yellow arrows). Note the persistent presence of the nasogastric tube (purple arrows) within the stomach for continued decompression. AP = anteroposterior

Seven days after the index operation, repeat imaging demonstrated a normal, non-obstructive bowel gas pattern (Figure 6). Clinical improvement was concordant with the histopathologic confirmation of viability in the remaining bowel following re-establishment of continuity.

This case highlights key radiologic steps for diagnosing pediatric intestinal obstruction when initial clinical and imaging assessments are nonspecific and emphasizes the need for prompt multimodal imaging, including dynamic fluoroscopic barium enema studies. Careful interpretation of these studies can facilitate timely surgical intervention and improved clinical outcomes.

## Discussion

Pediatric colonic volvulus is a rare but clinically critical cause of bowel obstruction. The transverse colon is among the least commonly involved segments, particularly when associated with underlying intestinal malrotation, which complicates both diagnosis and surgical management [1]. Early clinical recognition of pediatric volvulus is challenging in acute settings, and diagnostic delays can increase morbidity due to ischemic complications [7]. Underlying conditions, such as malrotation, chronic constipation, and Hirschsprung’s disease, and neurodevelopmental disorders like cerebral palsy may further increase the risk of volvulus and should heighten clinical suspicion and expedite imaging investigations [2,8].

Transverse colonic volvulus involves axial rotation of the colon around its mesenteric axis, resulting in intestinal obstruction and potential vascular compromise. This disruption of blood flow may progress to ischemic colitis, in which bowel injury arises from insufficient perfusion [9]. The gross and microscopic pathological features of transverse colonic volvulus vary according to the duration and severity of ischemia [10]. Acute, complete vascular obstruction can precipitate transmural necrosis of the bowel wall, whereas chronic ischemia is more often associated with persistent mucosal ulceration and marked submucosal fibrosis (Figure 6) [10,11].

On gross pathologic examination, ischemic colitis may present with mural and subserosal edema, stricture formation, scarring, and abnormal vascular patterns [12]. Early histopathologic findings typically include loss of mucin and surface epithelial cells, inflammatory cell infiltration, and hemorrhage with vascular congestion in the lamina propria. As the disease progresses, regenerating crypts and disrupted crypt architecture are observed, often interspersed with unaffected mucosa [11]. These ischemic alterations reflect the underlying hypoperfusion and tissue hypoxia that result from transverse colonic volvulus.

Advanced diagnostic imaging plays an indispensable role in confirming the diagnosis and guiding surgical intervention. The imaging manifestations of pediatric colonic volvulus vary by anatomical location and the degree of bowel rotation. While sigmoid volvulus frequently produces classic radiographic signs, transverse and proximal colonic volvulus often lack such features, complicating assessment. In these cases, contrast enemas and computed tomography (CT) are essential adjuncts to initial radiographs, enhancing diagnostic sensitivity and providing detailed anatomical localization [13].

Plain abdominal radiography serves as a pivotal initial diagnostic tool, but specificity is limited. Diagnostic accuracy for pediatric colonic volvulus may be as low as 22% due to overlapping features with other obstructive conditions [13,14]. Therefore, radiography functions primarily as a screening tool to detect generalized obstruction rather than confirm volvulus, reinforcing the need for additional modalities.

Ultrasound is also used as a rapid initial modality in pediatric bowel obstruction evaluation. Its primary role is to exclude competing diagnoses, such as intussusception, in undifferentiated patients. Direct diagnostic capability for colonic volvulus is limited, as visualization of signs such as the mesenteric whirl sign is inconsistent [15,16].

Contrast-enhanced dynamic barium enema fluoroscopic study remains the most reliable problem-solving modality and may confirm volvulus. Similar to adult cases, in which the tapered narrowing or “beak” sign is well-described, pediatric cases demonstrate high diagnostic success. Dynamic barium enemas may also achieve detorsion in some cases, but definitive surgery is generally required due to the high risk of recurrence [4].

Surgical intervention is often mandatory in pediatric colonic volvulus because of the rapid progression to ischemia and necrosis. In sigmoid volvulus, initial management may involve non-surgical techniques such as flexible sigmoidoscopy or radiologic detorsion under general anesthesia. However, when these approaches fail or when ischemia is evident, definitive resection becomes necessary. Options range from colopexy to sigmoid resection-performed laparoscopically or converted to open surgery when massive dilatation is encountered-to more extensive procedures, including left hemicolectomy or total colectomy with temporary ileostomy or pouch-anal anastomosis. The latter is particularly indicated when underlying conditions, such as Hirschsprung’s disease or chronic intestinal pseudo-obstruction (CIPO), are suspected. In rare cases of proximal colonic involvement, such as transverse colon volvulus, surgical detorsion combined with colostomy or colopexy is recommended [2]. Preoperative evaluation, including histologic assessment to exclude Hirschsprung’s disease and other congenital anomalies, along with multidisciplinary follow-up, is critical for optimizing outcomes and preventing recurrence. This comprehensive strategy underscores the necessity of a flexible, prompt, and individualized surgical approach to these challenging cases.

## Conclusions

This case underscores the need to maintain a high index of suspicion for the rare occurrence of mid-transverse colonic volvulus when evaluating the acute abdomen in pediatric patients. Imaging modalities, such as plain radiography, ultrasound, and particularly fluoroscopy-guided barium enemas, play crucial diagnostic roles, providing essential information for early recognition and guiding timely surgical planning. Future research directed towards developing standardized pediatric-specific imaging algorithms and further evaluating the role of advanced modalities, such as magnetic resonance imaging, is warranted. Such efforts may improve outcomes, particularly in children who cannot undergo dynamic fluoroscopic studies.
